# Preparation and characterization of general-purpose gelatin-based co-loading flavonoids nano-core structure

**DOI:** 10.1038/s41598-019-42909-0

**Published:** 2019-04-24

**Authors:** Xiaoqing Song, Kang Gan, Shuang Qin, Liang Chen, Xiuju Liu, Tianjie Chen, Hong Liu

**Affiliations:** 1 Department of Oral Comprehensive Treatment, School and Hospital of Stomatology, Jilin University, 1500 Qing Hua Road, Changchun, 130021 P.R. China; 2 Department of Stomatology, the First Affiliated Hospital of Zhengzhou University, Zhengzhou, 450000 P.R. China

**Keywords:** Drug delivery, Drug delivery, Drug delivery, Drug delivery, Drug delivery

## Abstract

Flavonoids (FLAs) possess anti-cancer, anti-viral, anti-bacterial, and anti-oxidant properties. In this study, gelatin nanoparticles (GNPs) with controllable surface potential and diameter was prepared through a modified two-step desolvation. Two well-known flavonoids, namely, low-molecular weight Genistein (GEN) and high-molecular weight Icariin (ICA), were adsorbed onto the surface of GNPs (FLA@GNPs). The characteristics of GNPs and the main parameters affecting flavonoid adsorption were studied to evaluate the adsorption capacity and structural stability of FLA@GNPs. Furthermore, co-adsorption of GEN and ICA was detected. The adsorption mechanism of GNPs with FLA was further discussed. Results showed that the low-molecular weight GEN could be effectively adsorbed by GNPs, and their entrapment efficiencies were over 90% under optimized conditions. The total drug loading of the co-adsorbed FLA@GNPs was significantly higher than that of the single drug loaded (GEN or ICA). GEN@GNPs could maintain its structural stability under acidic conditions (pH = 2) at room temperature (25 °C). This protective function enables both ICA and GEN to be bioactive at room temperature for at least 180 days. The characteristics of GNPs adsorption indicate that the hydrogen bonding theory of the combination of gelatin molecules with polyphenols cannot sufficiently explain the binding of GNPs with polyphenols. FLA@GNPs is a promising general-purpose gelatin-based co-loading preload structure with simplified operation and storage condition.

## Introduction

Flavonoids (FLAs) are natural polyphenols that widely exist in foods (fruits, vegetables, and teas) and most medicinal plants^1^. FLAs were originally identified as compounds containing 2-phenyl-chromone, while nowadays, they are referred to as having a common structure consisting of two aromatic rings linked by 3 carbons, mainly existing in the form of glycoside or aglycone^2^. More than 10,000 FLAs with a broad range of biological activities, i.e. anti-cancer, anti-viral, anti-bacterial and anti-oxidant, have been characterized over the past few decades^3,4^. However, the clinical applications of FLAs are limited, due to its chemical instability, low bioavailability, short half-life, poor compatibility with polymers, tendency to be discharged by the protein pump, and the sensitivity to gastric acid by oral administration^5–8^. Therefore, the modifications of the functional groups of natural FLAs have long become a research hotspot^9^.

To improve the bioavailability of FLAs, they have been modified through deglycosylation, glycosylation, acylation, and synthesis of metal complexes^10,11^. Although the relationships between the chemical structures and the biological activities of FLAs have been studied extensively, the complexity of the structures and the large quantity of interaction sites of FLAs impedes the researches on the structure-activity relationships^12–14^. Moreover, the types and the efficiencies of the methods should be considered during chemical modifications, and the changes of bioavailability and biological activities of FLAs after chemical modifications require further verification. The mechanism underlying the complex formation of metal ions remains unclear^15,16^. Therefore, to protect the organic functional groups and improve the bioavailability of FLAs, a safe and reliable modification method is needed.

Encapsulating functional components is a promising alternative way to modify FLAs, which efficiently prevents chemical degradation during processing and storage. Moreover, the stability of the encapsulated compounds could be enhanced by preventing enzymatic metabolism and thermal- or light-degradation^17^. In previous studies, the bioavailability of many natural compounds could be substantially enhanced by polymer materials, liposomes, and emulsions. Many bioactive agents, such as resveratrol, luercetin, curcumin, and vitamin C, have been loaded into delivery systems to improve water solubility, chemical stability, and bioavailability^18–21^. However, the loading of FLAs into the delivery systems is still limited, due to the crystalline structure of FLAs makes its dispersion in the carrier system less homogeneous, resulting in the higher initial burst release and the low encapsulation efficiency^22,23^. Moreover, the demand for loading two or more kinds of FLAs increases the difficulty of processing^24^.

Liposome-based materials are among a few natural biomaterials that can co-entrap FLAs and have been used in the preparations of many kinds of delivery systems^25^. Despite their good prospects, the high preparation cost and the difficulty of scale-up limit the applications of these materials^26^. In addition, preloaded composites require controllable surface characteristics to adapt to the preparations of specific delivery systems. For instance, platelet membrane needs neutral or negative surface potential to enhance its formation of the correct surface for the nanoparticle delivery systems^27^, while the delivery systems prepared from poly(lactide-co-glycolide) (PLGA) needs a positive surface potential to adapt to its entrapment efficiency and drug loading demand^28^. Gelatin is a water-soluble macromolecular chain extracted from collagen. Its hydrophobic and hydrophilic parts tend to migrate to the surface, thereby reducing the surface tension of the system and forming the same charge. The surface potential and the diameter can be effectively changed by adjusting the pH during particle formation. Owing to its high biocompatibility and biosafety, gelatin of low molecular weight has been used in the field of drug loading for more than 30 years. In addition to its thermal stability and potential disinfection properties^29,30^, gelatin can also protect the somatic cells from being destroyed *in vivo*, and gelatin nanoparticles has been used to deliver phytochemical or flavonoid^31,32^. Thus, in our study, we prepared gelatin-based nanocomposites (GNPs), which could load FLAs (FLAs@GNPs). It has been reported that the combination of polyphenols and gelatin depends on the molecular weight of the polyphenols. When the molecular weight is above 500, polyphenols can form a complex with gelatin^33,34^.

Genistein (GEN) and Icariin (ICA) are well-known FLAs, which have different sources, structures, and physicochemical properties^35^. GEN, an active isoflavone in soybeans and soy-based products, is almost insoluble in water, and has an average molecular weight of 270.24. ICA is a prenylated flavonol glycoside, which is isolated from Epimedium herb, and is one of the main effective components of the Epimedium herb. It is soluble in water and organic solvents, and has an average molecular weight of 676.65. It has been reported that both GEN and ICA could be loaded onto gelatin-based systems for the hydrophilicity or bioactivity improvement^36–38^. However, both electrospun poly (ε-caprolactone) PCL/gelatin nanofiber membrane and bioactive glass carrier need the assistant of other materials for icariin loading, leading to a relatively low loading capacity. Furthermore, researches do not cover co-loading flavonoids and long-term preservation.

**Figure 1:**
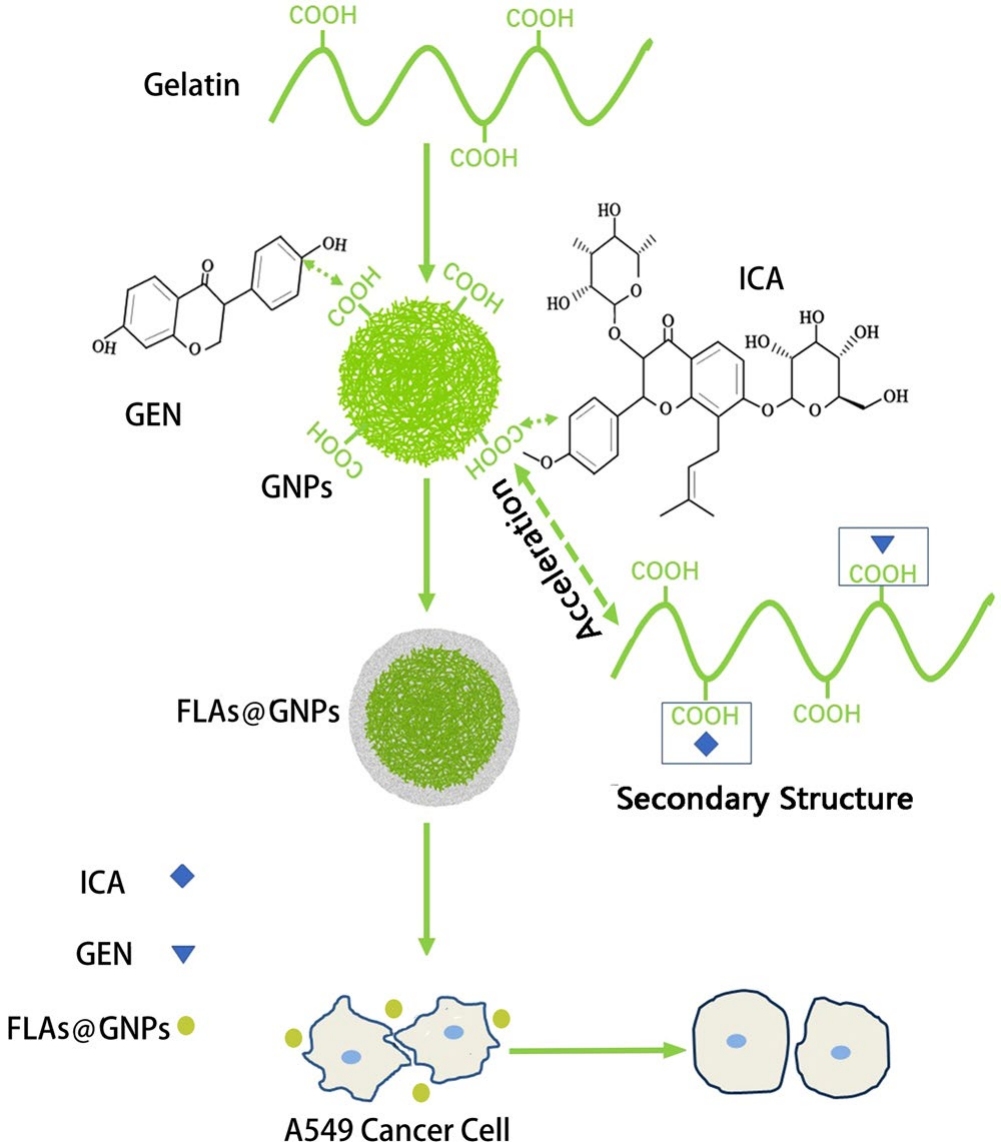
Scheme of the preparation of gelatin nanoparticles containing FLAs (ICA and GEN) and the effect on cancer cells.

In our study, to obtain a kind of preload structure, whose surface potentials, diameters, and the numbers of drugs can be changed to cope with the demand of different delivery systems, we firstly prepared GNPs, followed by coating with hydrophobic drug GEN with low molecular weight and hydrophilic drug ICA with high molecular weight^39^, as shown in Fig. 1. We detected the structural stability of nanocomposites, and optimized the main parameters affecting GEN and ICA adsorption onto GNPs. We also analyzed the effects of FLAs co-adsorbed on GNPs and the long-term biological activities. The adsorption mechanism of FLAs on GNPs was discussed. Our study demonstrated that FLA@GNPs are a kind of promising general-purpose gelatin-based co-loading preload structure, which could be applied to the delivery systems^40^.

## Results

### Preparation and characterization of GNPs

Two-step desolvation method^27,41^ was used to fabricate this controlled release system, with the pH at the second stage of nanoparticle preparation varied from 2 to 5. At pH ≥ 4.5, the gelatin solution was prone to precipitate immediately, rather than forming a nanosuspension. At pH < 2.5, the time for the conversion of the gelatin solution was prolonged, and the gradually emerging filamentous structure rapidly assembled into a precipitate.

A suspension was obtained at pH 2.5–3.5 with uniform and stable nanoparticle size. The minimum hydrodynamic diameter was 231 nm, when pH was 3.5 (Table 1). As the decrease in pH, the hydrodynamic diameter of the nanoparticle was increased to 275 nm at pH 3.0 and 257 nm at pH 2.5. The nanoparticle hydrodynamic diameter was increased to 391 nm when the pH was 4.0. These results were partly consistent with the publications^42,43^. At pH 2.3–3.8, the viscosity of gelatin solution suddenly decreased on the second desolvation stage. With the increase in pH, the solution of GNPs slightly changed from white to pale yellow.

**Table 1:** Physical properties of gelatin nanoparticles.

PH	Z-average (nm)	Half-width (nm)	PDI	Surface potential(e)	H-diameter (nm)	D-diameter/z-average (%)	w_D_ /w_H_ (%)
2.5	257	68.6	0.071	+10.69	118	45.9%	26.2%
3.0	275	77.3	0.079	+3.08	121	44.0%	24.5%
3.5	231	74.0	0.104	+29.61	106	45.8%	23.1%
4.0	392	147.0	0.16	+7.96	224	57.1%	28.7%

Z-average represents the hydrodynamic diameter. Half-width, PDI, and surface potential were detected using the Nano Brook 90plus PALS instrument. H-diameter was detected through SEM and AFM, representing the diameter of the GNPs after being freeze-dried. D-diameter/z-average represents the ratio of freeze-dried diameter to the hydrodynamic diameter of GNPs. *W*_*D*_ represents the weight of GNPs after lyophilization. *W*_*H*_ represents the weight of GNPs before lyophilization.

**Figure 2:**
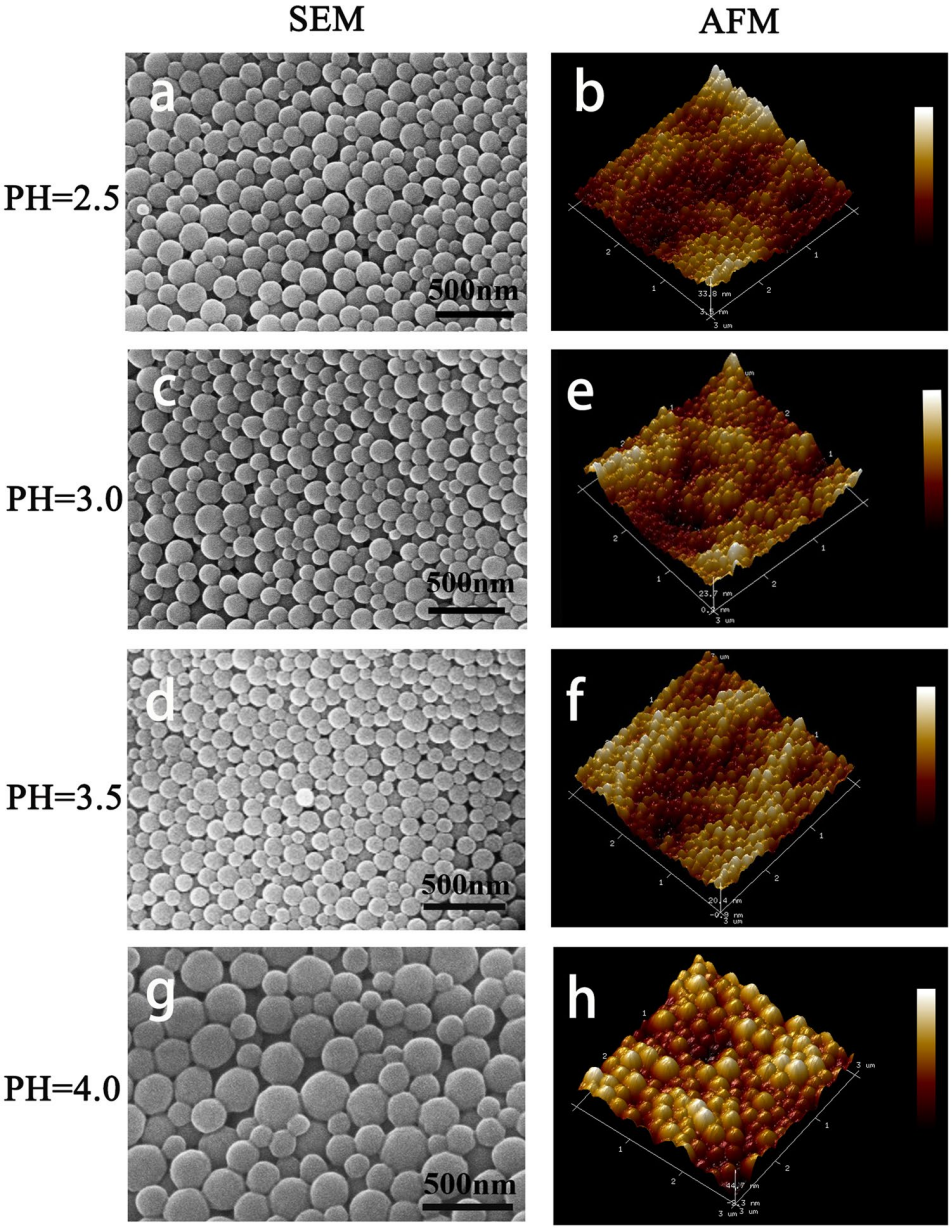
SEM and AFM images of GNPs from pH 2.5–4.0.

The shape of GNPs in water was ellipse or fusiform, according to the hydrodynamic diameter and width shown in Table 1. After being dehydrated through lyophilization, the diameters of GNPs had significantly decreased (P < 0.01). Furthermore, the surface potentials of GNPs were negatively associated with its diameter. The surface of GNPs showed a slight collapse after lyophilization by SEM and AFM images, and the weight of the particles decreased significantly, compared with those before lyophilization (P < 0.01) (Table 1). The GNPs exhibit a fusiform or ellipse structure and the diameter to width rate is 6:1 after lyophilization (Fig. 2b,e,f,h). The weight of GNPs was reduced from 20–30 mg to 5–6 mg after lyophilization (Table 1).

### FLAs adsorption onto GNPs

**Figure 3:**
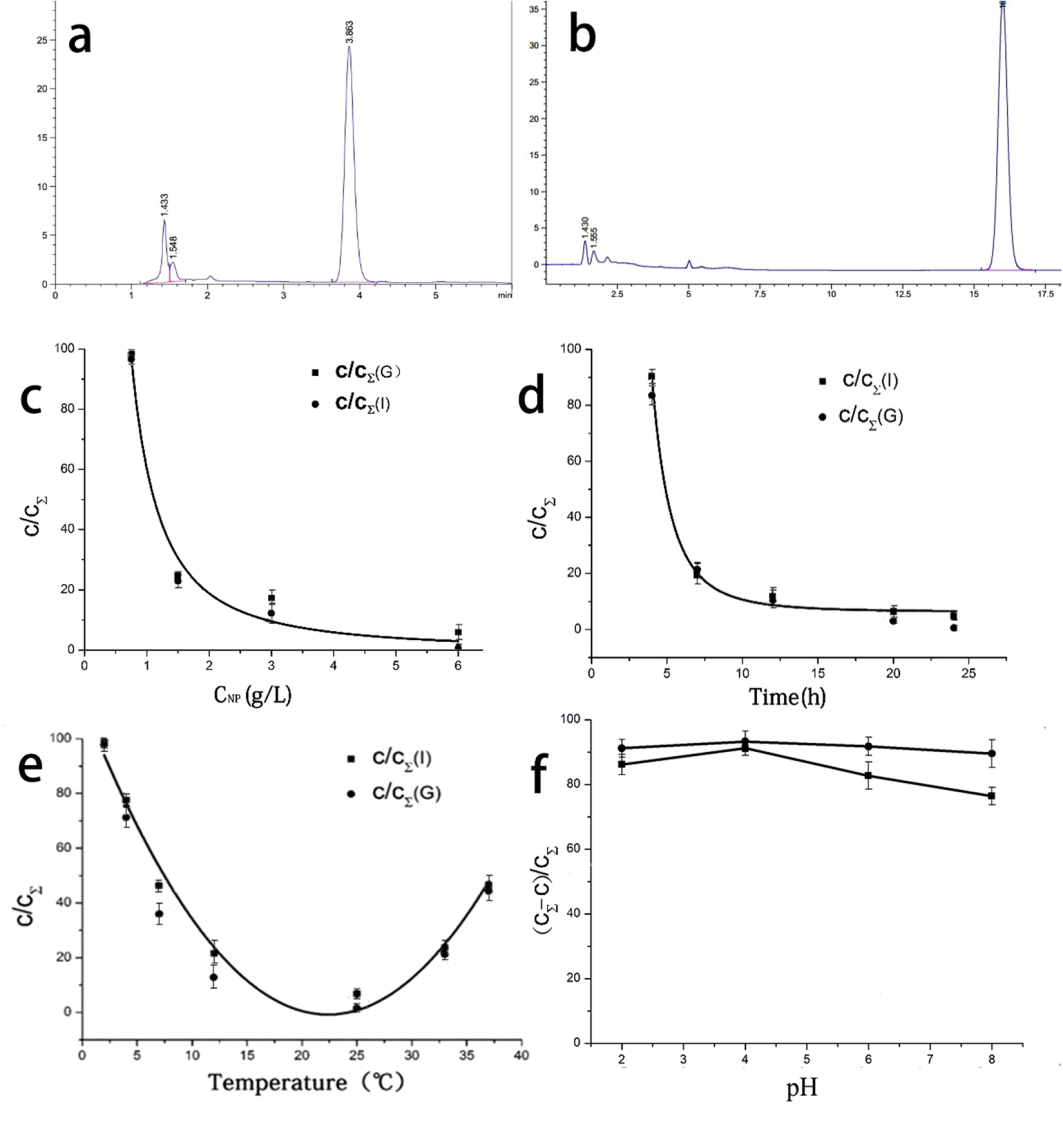
HPLC chromatograms and operating conditions described in the materials and methods section (means ± SDs). (**a**) GEN standard substance in the methanol aqueous solution detected by HPLC; (**b**) ICA standard substance in the methanol aqueous solution detected by HPLC; (**c**) the relationship between the concentration of nanoparticles and the percentage of free FLAs in the suspension; (**d**) the relationship between the time and the percentage of free FLAs in the suspension; e: the relationship between the temperature and the percentage of free FLAs in the suspension; f: the relationship between pH and the percentage of free FLAs in the suspension. C is free FLAs in suspension after loading GEN (G) or ICA (I) on GNPs. C_Σ_ is the total concentration at the beginning of the reaction.

The adsorption capacity of FLAs increased as the increase in GNP concentration (Fig. 3c). The adsorption capacity of FLAs exceeded 90% when the GNP concentration exceeded 3.0 g/L, and the characteristics of adsorption indicated the profile of allometric growth. FLAs adsorption was closely associated with the reaction temperature and the incubation time, and the adsorption exhibited a non-linear profile with exponential characteristics (Fig. 3d,e). When the temperature exceeded 20 °C or the incubation time exceeded 24 h, the adsorption capacity of GEN and ICA on GNPs exceeded 90%. GNPs maintained a high adsorption capacity at pH 2–8 (Fig. 3f).

The amount of ICA can reach 51.3% of the mass of the nanocomplex solid material. The GEN loading was approximately lower than 34.7%. The adsorption capacity of ICA was significantly higher (P < 0.05) than that of GEN, possibly because ICA has more -OH group than GEN. Excessive ICA and GEN contents were simultaneously placed into the nanosuspension with the highest drug loading of 46.1% and 27.2%, respectively. The total amount of FLAs was 73.3% of the total mass, which was higher than that for any single FLA.

### Stability analysis of nanocomposites

After FLAs were adsorbed on the GNPs to form nanocomposites in deionized water (DI water), considerable amount of precipitations existed after one month of storage in the refrigerator at 4 °C. The precipitates could not be dispersed through ultrasonication. Meanwhile, lyophilized nanocomposites formed at pH 3.5 were stored at room temperature for 1, 15, 30, 90, and 180 days, with the average diameters of 106.2, 108.3, 112.1, 114.2, and 113.2 nm, respectively (Table 2). Moreover, the loading capacities of ICA or GEN in the nanocomposites were stable. The detectable contents of loaded FLAs and the diameters of nanocomposites fluctuated in a small range during the 180 days, as shown in Table 2. This finding indicates that the lyophilized nanocomposites could be preserved for at least 180 days at room temperature.

**Table 2:** Summary of diameter and drug loading capacity of FLA@GNPs after different days stored at room temperature.

Time (D)	Diameter (nm)	DC (ICA) (%)	DC (GEN) (%)
1	106.2	68% ± 1.2	43% ± 0.8
15	108.3	65.8% ± 0.7	42.7% ± 1.3
30	112.1	65.5% ± 2.1	42.3% ± 0.3
90	114.2	61.7% ± 1.4	41.9% ± 1.7
180	113.2	53.6% ± 2.3	41.2% ± 1.1

### *In vitro* cytotoxicity of FLAs@GNPs

**Figure 4:**
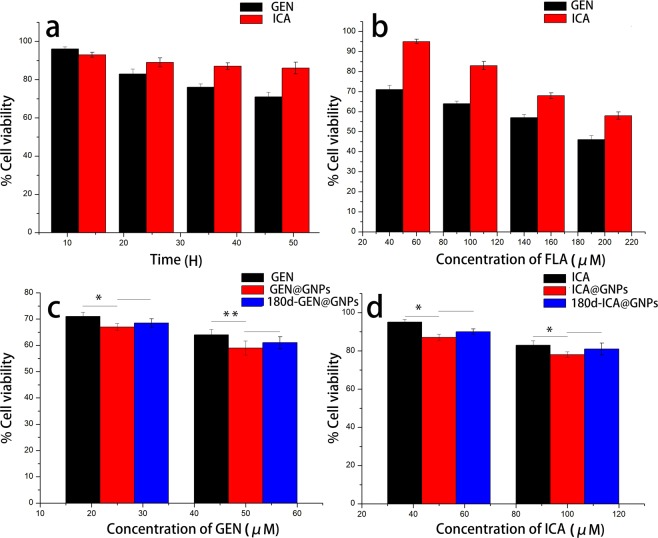
Effects of GEN and ICA on cell viability of A549 cell line by CCK-8 assay. (**a**) Cells were treated with 50 μM GEN or 100 μM ICA for 12, 24, 36, and 48 h; (**b**) A549 cells were treated with 50, 100, 150 and 200 μM of GEN or ICA for 48 h; (**c**) Cells were treated with GEN, GEN@GNPs, or lyophilized GEN@GNPs after stored at room temperature for 180 d (180d-GEN@GNPs) for 48 h, which were equivalent to 25 and 50 μM, respectively; (**d**) A549 cells were treated with formulated ICA, ICA@GNPs, or lyophilized ICA@GNPs after stored at room temperature for 180 d (180d-ICA@GNPs) for 48 h, which were equivalent to 50 and 100 μM, respectively. Data were obtained from four independent experiments. All values are expressed as means ± SDs. The experiment was repeated for three times.

To investigate the effects of GEN and ICA on the cell viability, we performed CCK-8 assay using A549 cell line^44,45^. Dose- and time-dependent cell viability was observed 48 h after treatments (Fig. 4a,b). Figure 4c,d showed that the *in vitro* cell viability of 180d-FLA@GNPs and FLA@GNPs exhibited cytotoxicity to A549 cells, both of which showed significantly higher cytotoxicity than FLA (P < 0.05). Meanwhile, no significant difference was observed between the 180d-FLA@GNP and FLA@GNP groups (P > 0.05).

**Figure 5:**
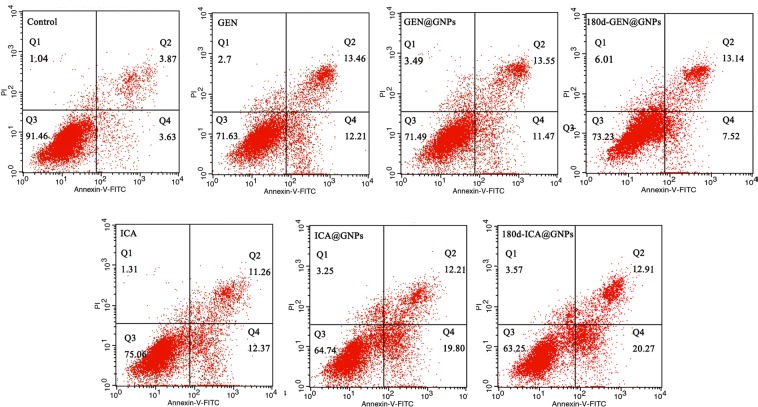
Flow cytometry analysis of A549 cells treated with 50 μM GEN@GNPs, 180d-GEN@GNPs, 100 μM ICA@GNPs, or 180d-ICA@GNPs for 48 h at 37 °C.

Further, we treated A549 cell line with GEN and ICA at the corresponding concentrations for 48 h, and assessed the apoptosis by flow cytometry (Fig. 5 and Table 3). The apoptosis of A549 cells treated with GEN or ICA was higher than the control group (P < 0.05). No significant difference was observed between the 180d-ICA@GNP and ICA@GNP groups (P > 0.05), which was consistent with the previous cytotoxicity result.

**Table 3:** The apoptosis rate in different groups ($\bar{{\rm{X}}}$ ± S, n = 4).

Groups	Apoptosis rate (%)		
	Q2	Q4	Q2 + Q4
Control	3.87 ± 0.24	3.65 ± 0.11	7.52 ± 0.14^a^
GEN	13.49 ± 0.67	11.75 ± 0.33	25.23 ± 0.80^b^
GEN@GNPs	14.43 ± 0.85	11.59 ± 0.42	25.93 ± 1.24^b^
180d-GEN@GNPs	13.43 ± 1.01	7.99 ± 0.33	21.42 ± 1.09^c^
ICA	12.01 ± 0.53	12.64 ± 0.65	24.65 ± 0.98^d^
ICA@GNPs	12.45 ± 0.94	19.63 ± 0.51	32.08 ± 1.43^e^
180d-ICA@GNPs	12.80 ± 0.72	19.48 ± 0.57	32.28 ± 0.88^e^

^*^Within the same column, the different superscripted letters indicate significant differences (*P < *0.05).

^a,b,c,d,e^Statistically significant differences between different groups.

## Discussion

Two-step desolvation method was used to fabricate GNPs, with the pH at the second stage of nanoparticle preparation varied from 2–5. Since the theoretical isoelectric point of gelatin (type A) is 6–7, the surface potential of GNPs is positive after being prepared under acidic conditions. To enhance the dispersion of nanoparticles, we used gelatin with relatively lower Bloom (240–270), as compared with the work of Tatsiana, who used 300 Bloom^41^. Smoluchowski aggregation theoretical analysis and experimental data suggest that the aggregation of gelatin is disordered at low temperature, whereas compact and ordered molecular clusters can be achieved at high temperature. Owing to the energy-barrier effect, the reaction should be conducted in a high-concentration solution^46^. Hence, some essential parameters such as initial of the first and second solution temperature, the content of glutaraldehyde, and the rate of addition were kept constant^47,48^.

The diameter of GNPs in liquid was higher than that of lyophilized GNPs, but GNPs in both status showed a similar length-to-width ratio. From the 3D images of AFM (Fig. 2b,e,f,h), the shape of GNPs tended to be in an ellipse or fusiform, exhibiting a curved shape like a circle, but with two slightly longer and flatter sides. Moreover, the similar length-to-width ratio at both status indicated that the volume and surface morphology of GNPs shrank at an equal ratio. Furthermore, the diameters of GNP were negatively associated with the surface potentials. This finding is possibly due to the surface migration of hydrophilic or hydrophobic amino acid sequence on the side chain of the gelatin molecule. A high zeta potential has the potential to resist aggregation of the nanoparticles, and vice-versa.

The adsorption of FLAs on gelatin mainly occurs through the formation of hydrogen bonds between phenolic rings and hydrophobic amino acids^49^; The concentration of GNPs and the temperature of reaction affected the FLAs adsorption. The peak adsorptive capacity of GNPs was achieved by increasing the temperature to 23 °C, and then it decreased with further increased temperature. The interaction between polyphenols and proteins is a reversible and multistage process^50^. It has been reported that the adsorptive capacity of GNPs was improved by increasing temperature^41^. This phenomenon may be attributed to the more exposed hydrophobic groups in the gelatin molecules with the increasing temperature, which increased the chances of collision with GEN and ICA. In the initial stage of adsorption, the increasing adsorption efficiency per unit time can be partly attributed to the exothermic process of adsorption reactions between GNPs and FLAs. The complex structure of polyphenols and proteins dissociates at a high temperature, and the protein returns to its free state from the bound state of the complex. Dissociation generally occurs beyond 80 °C^51^. Meanwhile, this bond could be broken at 40 °C. However, the dissociation temperature of FLA@GNPs is lower than the reported temperature. Hence, this composition is prone to dissociation at room temperature. Therefore, an efficient way of preservation is particularly necessary.

Time is another important factor of affecting FLAs adsorption, suggesting that the adsorption of ICA or GEN on GNPs is a slow process. Only 10.6% ICA and 16.48% GEN were adsorbed on GNPs in the first 4 h. The adsorption rate increased remarkably within the next 4 h to approximately 68.1% for ICA and 64.92% for GEN. The reaction between GNPs and FLAs was inactive at the initial time and became active when the reaction proceeds until all FLAs were adsorbed. The recommended reaction time was 15 min at room temperature^52^. The amount of adsorbed FLAs was below 10% in our study (Fig. 3d). According to Hagerman^53^, gelatin molecules could adsorb more polyphenols after 24 h of reaction at 4 °C, compared with that after 15 min of reaction at room temperature. However, our experiments showed that FLAs could hardly be adsorbed on GNPs at 4 °C even with a reaction time of over 48 h.

We hypothesized that the new structure formed by the reaction may significantly boost or activate the reaction as a feedback to the further adsorption. The third stage of time showed that GNPs did not maintain an active adsorption capacity. This finding could be attributed to the saturated binding sites of the GNPs.

The amount of ICA or GEN in the co-adsorbed nanocomplex was significantly less than that in the single-loading nanocomplex, i.e. ICA@GNPs or GEN@GNPs. Meanwhile, the maximum drug loading of the co-adsorbed gelatin nanocomposites was significantly higher than that of either single drug-loaded nanocomposites. This result indicated that the existence of different binding sites between ICA and GEN could be attributed to the combined binding sites on the surface of GNPs and the formed spatial structures, which increased the drug-loading capacity. The binding characteristics with polyphenol of the GNP nanostructure were significantly different from those of the gelatin molecules from pH 2–8. Therefore, the combination of GNPs and polyphenols cannot be explained only by the theory from gelatin molecules with polyphenols.

In our experiment, the increase in pH did not influence the amount of FLAs adsorbed onto the nanoparticles (Fig. 3f). Hence, the functional components of FLAs@GNPs can be effectively maintained at pH 2 in preparation with other carrier materials, thereby widening the scope of its application. The CCK-8 assay and flow cytometry results suggest that 180d-ICA@GNPs and ICA@GNPs caused cytotoxicity to A549 cells, which was higher than single ICA. Even though 180d-GEN@GNPs caused cytotoxicity to A549 cells, which was lower than single GEN, it was still significantly higher than that of the control group and maintained high cytotoxicity.

The GNPs changed the mechanism of the formation of polyphenol-protein complexes. The adsorption temperature and time showed significant changes. The newly formed gelatin-FLA structure accelerated the adsorption but required the participation of new surface groups of GNPs for this effect. Incorporating more than one kind of FLAs onto GNPs is possible. The nanocomposites could maintain biological activity after being stored at room temperature for 180 days. This characteristic is conducive to the storage and transport of FLAs. Moreover, solid preservation can improve the stability more effectively than liquid storage, and the reduced volume decreases the storage and transport costs. Our study presented a simplified fabrication process of a new drug loading system with good structural stability, low price, controllable surface potential and diameter, and capacity for co-loading. We reported the surface potential of size-controllable GNPs as a general pre-loading nanocomplex. This compound could potentially improve the compatibility of FLAs with other carrier materials with good structural stability and bioavailability. Low-molecular weight GEN and high-molecular weight ICA can be effectively loaded by GNPs simultaneously with high drug loading capacity. The FLA@GNPs nanocomplex could effectively maintain its biological activity over 180 days, thereby allowing transport and long-term storage at room temperature.

## Conclusions

Our current study presented a simplified fabrication process of a new drug loading system with good structural stability, low price, controllable surface potential and diameter, and capacity for co-loading. FLA@GNPs are a promising general-purpose gelatin-based co-loading preload structure and could be applied to the synthesis of many delivery system with simplified operation and storage condition, which are beneficial for its industrialization. Identifying new binding sites or new spatial conformations should be the focus of subsequent theoretical improvement.

## Materials and Methods

### Materials

For cell studies, we used an immortalized human lung adenocarcinoma epithelial cell line A549 (Department of Medical Genetics, School of Basic Medical Sciences, Jilin University). Dulbecco’s modified Eagle’s medium (DMEM), penicillin-streptomycin, trypsin, and fetal bovine serum were purchased from Sigma (USA). Cell Counting Kit-8 (CCK-8; Sigma, USA), acetone, anhydrous alcohol, GEN, ICA, gelatin from porcine skin (gel strength 250 g Bloom, Type A), glutaraldehyde, and acetone were purchased form Sigma (USA). A Zorbax® SB-C_18_ high-performance liquid chromatography (HPLC) column (5 μm; 4.6 × 150 mm) for FLA analysis was purchased from Agilent Technologies (USA). HPLC-grade solvents methanol and acetonitrile were purchased from Sigma-Aldrich and Fisher Scientific (USA). All other solvents and chemicals used were in high-grade purity. Fluorescence inverted microscope (Olympus, CKX41, Japan), desktop centrifuge (Eppendorf, Mini Spin, German), JB-2 constant temperature magnetic stirrer (Shanghai Lei Xin Instrument Co., Ltd., China), KQ-100DA ultrasonic oscillators (Kunshan Ultrasonic Instrument Co., Ltd. China), transmission electron microscope (TEM; JEM-1011, Japan), SEM (Hitachi S-4800, Japan), and AFM (VEECO Dimension icon, USA) were used.

### Nanoparticle preparation

GNPs were prepared using a modified two-step desolvation method^27,41^. Briefly, 0.5 g of gelatin was dissolved in 10 mL of DI water by gently heating to 60 °C. Then, 10 mL of acetone was rapidly added to the solution, which was shaken slightly. The precipitate was redissolved in 10 mL of water by light heating to 60 °C, and the pH was adjusted using HCl and NaOH to 2.0–5.0. Under constant stirring at 500–1000 rpm at 40 °C, 35 mL of acetone was slowly added within 7 min. Thereafter, 0.04 mL of 50% glutaraldehyde was added while stirring, with the stirring continued for 1 h. After 8 h of incubation at room temperature, the resulting GNPs suspension was placed in plastic tubes and stored in a refrigerator at 4 °C until use.

### FLA adsorption onto nanoparticles

The amount of GEN or ICA adsorbed onto GNPs from methanol solution was compared at different GNP concentration, time, temperature, and pH values. The prepared suspension was divided into 2 mL aliquots, and the GNPs were separated from the supernatant through centrifugation at 6,500 rpm for 20 min, followed by washing with 75% aqueous acetone for three times. Finally, the content of each tube was resuspended in 1 mL of aqueous solution to concentrate the sample.

One mL of 0.25–2.5 mg/ml GEN or ICA was mixed with 1 mL of nanoparticle suspension at 2, 7, 12, 24, 33, and 37 °C. The pH was adjusted using HCl or NaOH. After 1, 6, 12, 24, and 25 h, the FLA-loaded nanoparticles were separated from the supernatant through centrifugation at 6,500 rpm for 20 min. The loaded nanoparticles were then dried at room temperature until use. HPLC was used to test the concentration of FLAs in the remaining suspensions. The amount of FLAs in the nanocomposite can be calculated using the standard curve equation.

### Characterization of nanoparticles

Surface morphology was assessed using SEM (S-4800, Hitachi, Japan) and AFM (Dimension Icon, VEECO, USA). Sample surfaces were spattered with a 5 nm-thick layer of iridium to enhance the image quality. The samples were prepared on a freshly cleaved silicon chip. Nanoparticle hydrodynamic diameters and potentials were measured by Nano Brook 90plus PALS(Brookhaven Instruments Corp, USA) instrument in water. For the measurements, 0.2 mL of nanoparticle suspension was redispersed in 4 mL of water. The diluted solutions were stored for approximately 24 h before measurements to determine the isoelectric point of GNPs. Dried GNPs containing FLAs were obtained using an EYELA FDU-2200 (EYELA, Tokyo Rikakikai Co., Ltd.) freeze drier, and the samples were frozen in liquid nitrogen. The quality of solid residue in the GNPs sample was analyzed using an accurate electronic balance. Then, 2 mL of nanoparticle suspension was washed three times with 75% acetone, centrifuged, resuspended in 1 mL of aqueous solution, and lyophilized. Three independent experiments were performed, and the averages of the results were obtained.

GEN and ICA concentrations in the sample were measured by HPLC. A calibration curve was constructed at ultraviolet absorption wavelengths of 262 and 270 nm as a function of GEN and ICA contents in phosphate buffer saline (PBS) in the linear range. This curve was used as the multipoint working curve. The HPLC conditions were as follows: column, Zorbax® SB-C_18_ (5 μm; 4.6 × 150 mm); mobile phase, 55% A + 45% D–60% A + 40% D (A, H_2_O + (0.1–0.2) % H_3_PO_4_; D, methanol); flow rate, 1.0 mL/min; and column temperature, 30 °C.

### Stability test of FLA/GNP nanocomposites

The prepared suspension was centrifuged, the supernatant was discarded, and the same amount of aqueous solution was added. The suspension was stored in a refrigerator at −80 °C for 24 h before lyophilization, followed by storage at room temperature for 1, 15, 30, 90, and 180 days. A small amount of solid powder was obtained and placed on the surface of conductive cloth tape. Gold particles were sprayed on the surface of composites in vacuum for 5 nm. SEM observation was conducted, and the particle size distribution was calculated using the Nanometer 1.2 software.

### *In vitro* cellular toxicity and apoptosis of FLA@GNPs

A549 cells were trypsinized and seeded at 4 × 10^3^ cells/well in 96-well plates. GEN and ICA were designed as the control groups, whereas the GEN@GNPs, ICA@GNPs, 180d-GEN@GNPs and 180d-ICA@GNPs were the experimental groups. 180d-FLA@GNPs, FLA@GNPs, and FLA were at equivalent concentrations of 25 and 50 μM for GEN and 50 and 100 μM for ICA. After 24 h of cell seeding, 200 μL media in each group was added and incubated for an additional 48 h. Subsequently, 10 μL of CCK-8 solution (5 g/L; dissolved in PBS) was added. Plates were incubated for additional 4 h. The optical density for each well was measured using a microculture plate reader (Bio-Tek, USA) at 450 nm wavelength. Cell viability was estimated according to the following equation:

$$ \% \,{\rm{of}}\,{\rm{control}}={{\rm{OD}}}_{{\rm{Treated}}}/{{\rm{OD}}}_{{\rm{Control}}}\times 100 \% $$

(OD _Control_ was obtained in the presence of FLAs, and OD _Treated_ was obtained in the presence of FLA@GNPs).

The relative percentage of apoptotic and necrotic A549 cells was assessed through flow cytometry. A549 cells were seeded into six-well plates at an initial cell density of 1.5 × 10^5^ cells/cm^2^ and co-cultured with free GEN (50 µΜ), ICA (100 µΜ), GEN@GNPs, 180d-GEN@GNPs, ICA@GNPs, or 180d-ICA@GNPs for 48 h at 37 °C. Then, the cells were resuspended in Annexin V binding buffer and stained with Annexin V-FITC/PI Apoptosis Detection Kit (Key GEN Bio-TECH, Jiangsu, China) before analysis through flow cytometry.

### Statistical analyses

Results were represented as mean ± standard deviation (*n* = 4 or 3). The data were analyzed through one-way ANOVA, and differences were considered as statistically significant at P < 0.05.
